# Characterization of nsp1 Binding to the Viral RNA Leader Sequence of Severe Acute Respiratory Syndrome Coronavirus

**DOI:** 10.1021/acs.biochem.4c00078

**Published:** 2024-05-08

**Authors:** Jonathan L. Cromer, Laurie F. Melton, Kaitlin M. Caughman, Anita Nag

**Affiliations:** † Natural Sciences and Engineering, USC Upstate, Spartanburg, South Carolina 29303, United States; ‡ Department of Chemistry, Clemson University, Clemson, South Carolina 29634, United States; § Harvard University, Cambridge, Massachusetts 02138, United States

## Abstract

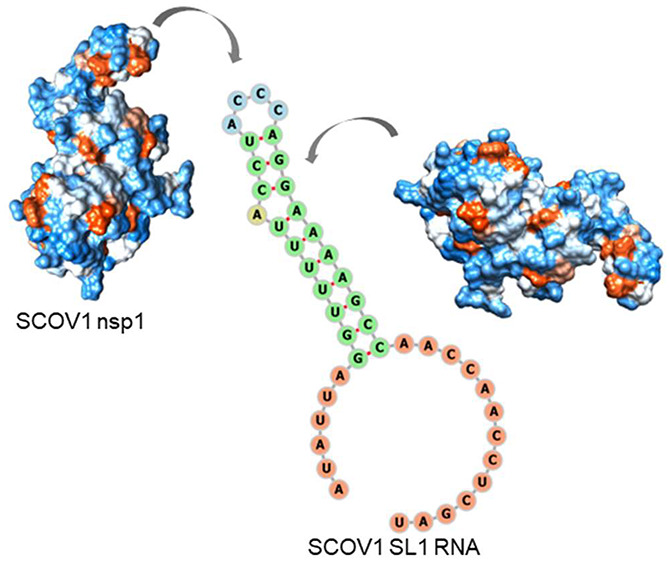

Nonstructural protein 1 (nsp1) of the severe acute respiratory syndrome coronavirus (SCOV1 and SCOV2) acts as a host shutoff protein by blocking the translation of host mRNAs and triggering their decay. Surprisingly, viral RNA, which resembles host mRNAs containing a 5′-cap and a 3′-poly(A) tail, escapes significant translation inhibition and RNA decay, aiding viral propagation. Current literature proposes that, in SCOV2, nsp1 binds the viral RNA leader sequence, and the interaction may serve to distinguish viral RNA from host mRNA. However, a direct binding between SCOV1 nsp1 and the corresponding RNA leader sequence has not been established yet. Here, we show that SCOV1 nsp1 binds to the SCOV1 RNA leader sequence but forms multiple complexes at a high concentration of nsp1. These complexes are marginally different from complexes formed with SCOV2 nsp1. Finally, mutations of the RNA stem-loop did not completely abolish RNA binding by nsp1, suggesting that an RNA secondary structure is more important for binding than the sequence itself. Understanding the nature of binding of nsp1 to viral RNA will allow us to understand how this viral protein selectively suppresses host gene expression.

In the past two decades, we experienced multiple zoonotic virus outbreaks, including SARS coronavirus 1 (SCOV1, 2002), MERS coronavirus (MERS-COV, 2014), and SARS coronavirus 2 (SCOV2, 2019).^1−3^ Both SCOV1 and SCOV2 possess a long (30 kb) single-stranded positive-stranded RNA genome.^3^ After entering the host cell, the viral genome undergoes translation by hijacking the host cell’s translational machinery.^4−6^ Both structural and nonstructural proteins are translated from viral RNA, and polyproteins (pp1a and pp1b) containing nonstructural proteins are processed by proteases to generate 16 nonstructural proteins (nsp1–16).^3^ Despite having sequence differences, nonstructural protein 1 (nsp1) of different coronaviruses (transmissible gastroenteritis virus or TGEV, MERS, and SARS) share a similar function of suppressing host gene expression.^7^ Moreover, deletion mutations of nsp1 severely impair viral activity.^8^ The nsp1 of SCOV1 and SCOV2 serves as a host shutoff protein by selectively blocking host mRNA translation and triggering mRNA decay while allowing viral RNA to escape this host shutoff mechanism.^6,9−11^ Thus, while viral RNA translation and viral protein synthesis continue, host mRNA translation is significantly inhibited. Recently generated cryo-electron microscopy-based models predict that nsp1 binds to the 40S ribosome, thereby precluding the mRNA binding site and inhibiting host mRNA translation.^12,13^ It is still puzzling to explain how viral RNA displaces nsp1 from the 40S ribosome’s mRNA binding site to overcome the inhibition. Moreover, if nsp1 blocks the mRNA binding site of the 40S ribosome, then it is difficult to explain how the 48S ribosomal complex formed in the presence of SCOV1 nsp1 and reporter RNA.^6^ Surprisingly, nsp1 can distinguish between host mRNAs and the viral RNA despite the latter’s resemblance to host mRNAs. Both host mRNA and viral RNA feature a 7-methyl guanosine cap at the 5′-end and a poly(A) tail at the 3′-end that protects RNA from exonucleases. Studies in SCOV1 proposed that the key to nsp1’s recognition of the viral RNA lies in the viral leader sequence.^14^ Viral genomic RNA and subgenomic RNAs carry a 100-nucleotide-long common leader sequence at the 5′-end. This sequence contains three stem-loop secondary structures of which deletion of the first stem-loop abolishes viral RNA’s ability to be protected from nsp1-mediated translation inhibition.^14^ Recent studies proposed that nsp1 in SCOV2 binds to the first stem-loop sequence (stem-loop 1 or SL1).^15,16^ This binding may outcompete nsp1’s binding to the mRNA binding pocket of the 40S ribosome allowing viral RNA to undergo translation.^9^ Molecular dynamic simulation with SCOV2 nsp1 predicted that the C-terminal flexible region of nsp1 binds to the loop-proximal area of stem-loop 1.^15^ SCOV1 and SCOV2 nsp1 proteins share 84% identity, while the viral leader sequences share structural homology. Since nsp1 in both SARS coronaviruses triggers host shutoff by similar mechanisms, we seek to characterize the RNA binding properties of SCOV1 nsp1 and compare this binding to that of SCOV2 nsp1.

A combination of molecular dynamic simulation and gel shift assay established the binding of SCOV-2 nsp1 to the corresponding viral leader sequence.^15^ In addition, computational modeling predicted that R124 and K125 residues in the C-terminus region of nsp1 play a key role in binding the loop-proximal site of SL1 of the viral leader sequence.^15^ Since SCOV1 and SCOV2 nsp1 proteins show 84% sequence identity and share the common function of selectively blocking host translation, we sought to characterize direct binding between SCOV1 nsp1 and the SCOV1 leader RNA sequence. Biotin-labeled 44-nucleotide-long RNA containing SL1 was incubated with purified SCOV1 nsp1 followed by the detection of complexes using a gel-shift assay coupled with chemiluminescence detection. Nsp1 binding resulted in the formation of a complex with slower mobility than free RNA, confirming nsp1–SL1 complex formation (Figure 1A, lanes 1 and 2).

**Figure 1:**
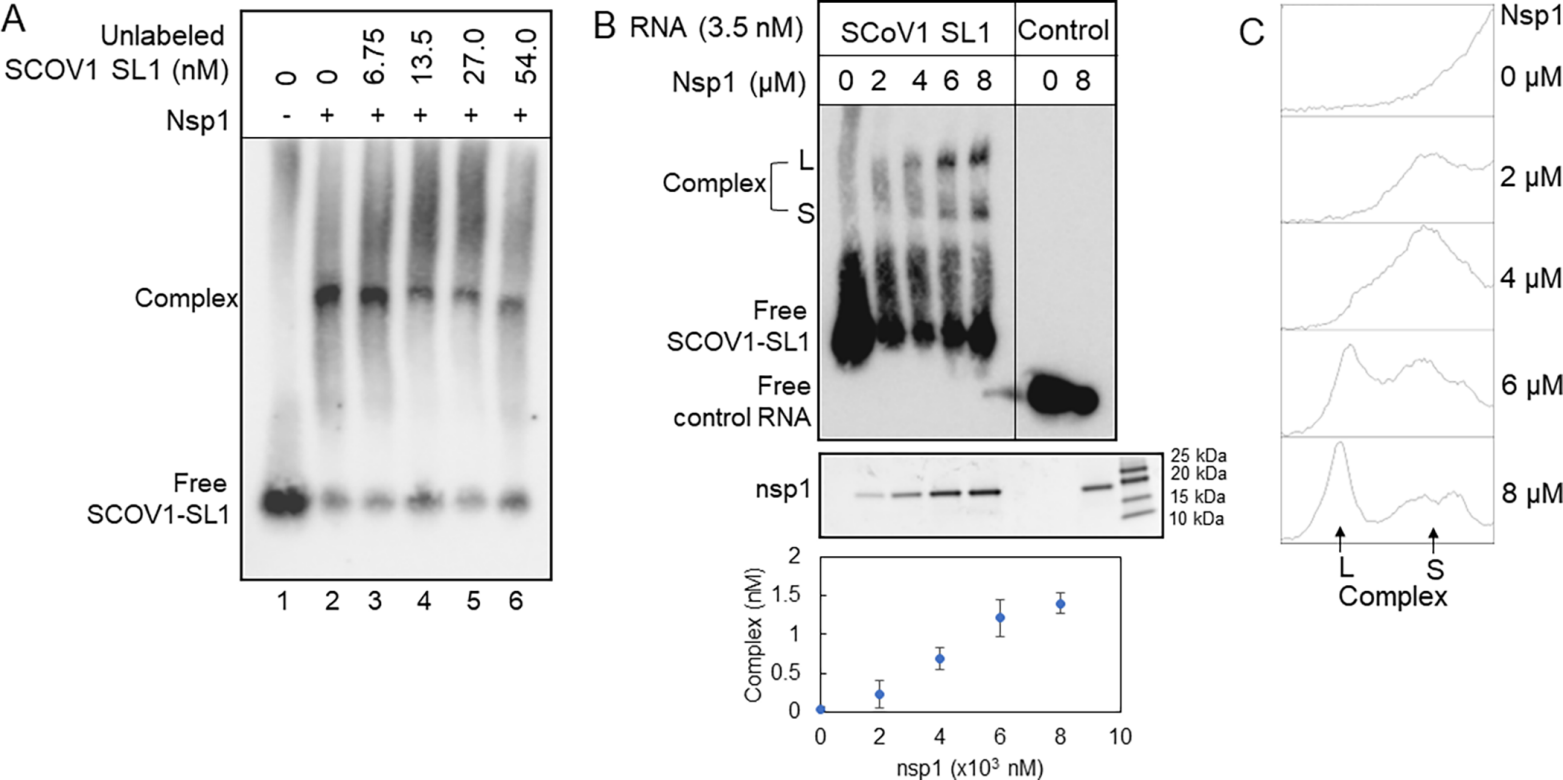
SCOV1 nsp1 binds the viral RNA leader sequence. (A) Gel-shift assay with the biotin-labeled viral RNA sequence containing the first stem-loop (SL1) of SCOV1. Prefolded RNA is incubated with 8 μM purified nsp1 followed by gel electrophoresis and transferred to a positive charge nylon membrane. Free RNA and complexes are identified using chemiluminescence. Sequence specificity of binding is detected by a gel shift assay using competitor unlabeled SL1. (B) An increase in concentration of nsp1 generates a slow-moving complex along with a fast-moving complex formed at a low concentration of nsp1. The control RNA is supplied with the EMSA kit (top). Nsp1 protein is visualized by Coomassie staining (middle). Complexes are analyzed by ImageJ and plotted with the increasing concentration of nsp1 from four (*n* = 4) separate experiments. The error bars represent standard deviation (bottom). (C) The S and L complexes from a repeat experiment are analyzed with ImageJ analysis, and complex intensity is plotted showing the formation of the L complex at a higher nsp1 concentration.

To establish the specificity of binding, we added an increasing amount of untagged competitor RNA (SL1) in the presence of a fixed concentration of the biotin-tagged RNA–nsp1 complex (Figure 1A, lanes 3–6). Results show a concentration-dependent decrease in the biotin-tagged RNA–nsp1 complex when unlabeled competitor RNA was used. When nsp1 was added to a control RNA (see the Materials and Methods), we did not observe any binding (Figure 1B). Moreover, we did not observe binding if the viral leader sequence was not folded prior to its incubation with nsp1 (data not shown). Next, we added an increasing concentration of nsp1 that resulted in a stepwise formation of at least two RNA-bound nsp1 complexes (Figure 1B). The dissociation constant for SCOV1 nsp1 (*K*_d_ = 1.40 ± 0.12 μM) was higher than the reported dissociation of SCOV2 nsp1 to SL1^15^ (*K*_d_ = 0.18 ± 0.01 μM). Even though both our experiment and Vankadari et al. used the same length SL1 RNA, there are some differences in the sequence and structures of SCOV1 and SCOV2 SL1 RNA (see below), which may contribute to the difference in binding.^15^

In contrast to the binding of SCOV2 nsp1 to its RNA, an increase in the concentration of SCOV1 nsp1 resulted in two separate band formations (Figure 1B). An ImageJ analysis of these bands shows that at a lower concentration of nsp1, a faster-moving or smaller complex (complex S) forms but gradually dissipates, while the slower-moving or larger complex (complex L) starts to accumulate at a high concentration of nsp1 (Figure 1C). We concluded that SCOV1 nsp1 binding to its corresponding RNA results in multiple complexes that may arise due to multiple copies of either nsp1 or RNA being engaged in binding. It is also possible that conformational change in RNA may generate complexes that have different mobilities in the native gel.

Once we established that SCOV1 nsp1 binds the viral RNA (SL1) directly, we sought to identify whether a specific sequence in the stem-loop is necessary for this interaction. To address sequence-specific binding, we performed a gel-shift assay with purified nsp1 and SL1 RNA containing stem and loop mutations. We created individual SL1 mutants in which we disrupted the stem 1b and 2b regions (Figure 2A, 22–27 CCCUUU) or replaced the ACCC sequence of the loop with GAAG (18–21 GAAG) or deleted the ACCC sequence completely (19–21 CCC deletion). According to RNAfold (http://rna.tbi.univie.ac.at/cgi-bin/RNAWebSuite/RNAfold.cgi), only 22–27 CCCUUU mutations significantly changed the stem-loop secondary structure while other mutations formed stem-loops that are similar to SL1. In addition, we weakened the 1a stem by creating a bulge (13–15 AAA) or replaced the sequence of the 1b stem with a sequence complementary to the 1a mutation to result in a perfect stem-loop structure (15–17GUA/22–25UAC). Finally, we replaced the entire loop proximal region with an alternative stem-loop sequence (15–25 GUAGAAGUAC). None of these mutations blocked nsp1’s binding to SL1 but changed the abundance of slow- and fast-moving complexes. Among all mutations, the mutations that diminished slow-moving complex formation are mutations 22–27 CCCUUU and 13–15 AAA (Figure 2B). These two mutations significantly changed the SL1 structure, as predicted by the RNA folding prediction software. We conclude that the structure of the stem-loop plays a more significant role in nsp1 binding than the exact sequence.

**Figure 2:**
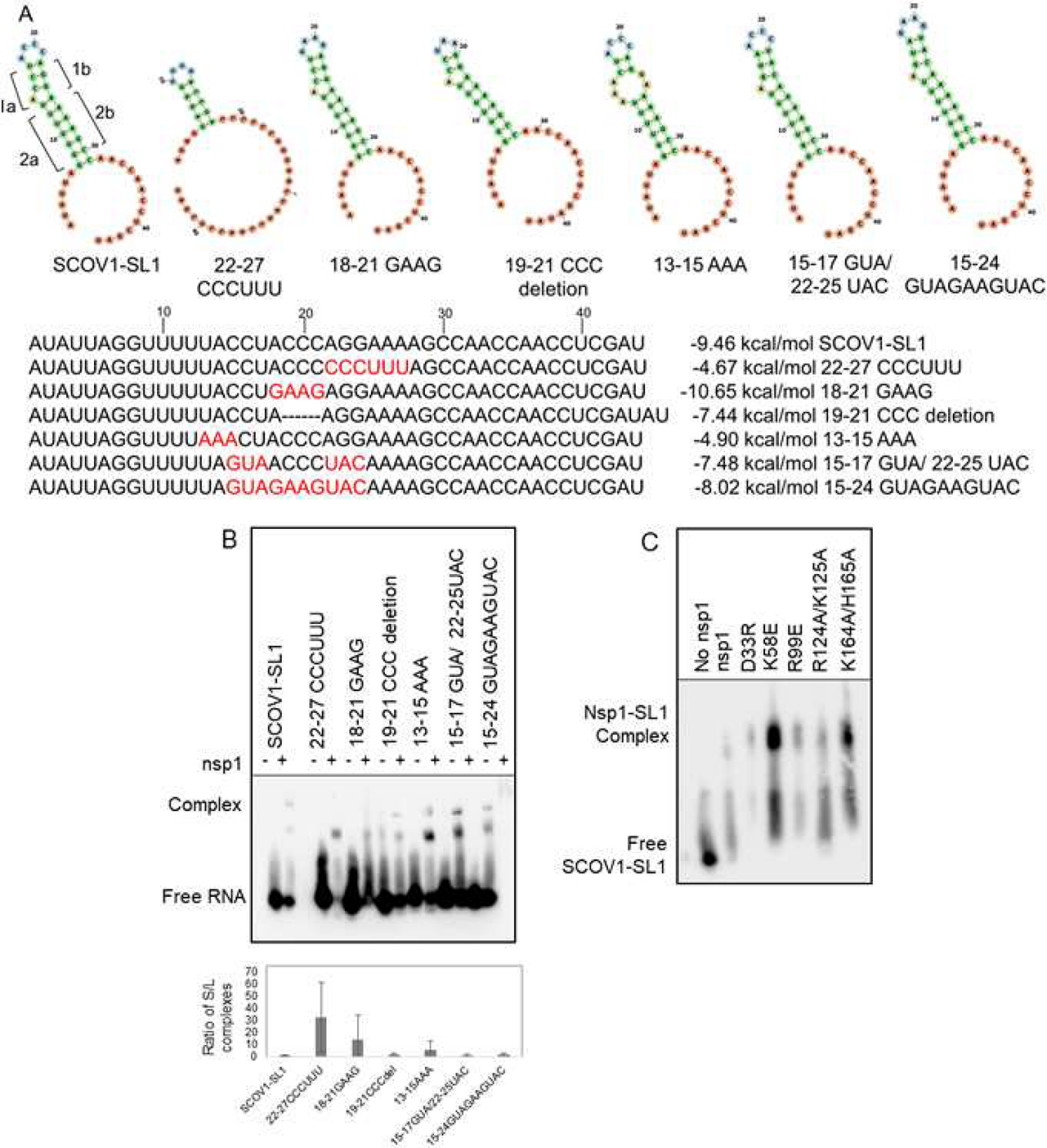
SCOV1 nsp1 binds to stem and loop mutations of SL1. (A) RNA secondary structures of SL1 and stem-loop mutants are analyzed in the RNA fold. (B) SCOV1 nsp1 binds to SL1 RNA even after stem and loop mutation. Gel-shift assays of biotin-tagged wild-type and mutant RNA-bound complexes of SCOV1 nsp1 (8 μM) are visualized using chemiluminescence. (C) SCOV1 SL1 binds to nsp1 mutants. Gel shift assay of mutant nsp1 (6 μM) and SL1 was captured by chemiluminescence.

In SCOV2, computational modeling supports an active role of R124 and K125 residues of nsp1 in RNA binding.^15^ Therefore, we sought to validate whether R124 and K125 residues of SCOV1 nsp1 are responsible for its binding to SL1 RNA by comparing its binding to that of wild-type nsp1. We also included the K164A/H165A mutation (inactive in translation suppression) and several other mutations (D33R, K58E, and R99E) with defects in the host shutoff function to test their binding to SL1 RNA.^17^ None of these mutations eliminated nsp1’s ability to bind SL1 RNA (Figure 2C). These results suggest that mutations used here do not participate directly (or individually) in RNA recognition and binding. Indeed, computational modeling of SCOV2 nsp1 and SL1 by Sakuraba et al. predicted multiple binding sites on nsp1 (S40, R43, K47, D75, R124, N126, H127, A131, H134, S135, Y136, and K141, either through the side chain or the backbone) with the loop and loop-proximal region of SL1.^16^ Authors predicted that multiple amino acid residues capable of H-bonding and ionic interaction (Arg43, Lys47, Arg124, and Asn126) may make independent contacts with the loop and its proximal region. Therefore, it is expected that the mutation of individual residues will not perturb the binding.

Next, we investigated if SCOV1 nsp1 also binds SCOV2 RNA by subjecting SCOV2 stem-loop 1 (SCOV2-SL1) to SCOV1 nsp1 binding followed by a gel-shift assay. We used SCOV2 nsp1 as a control. In addition, we used two separate mutations in SCOV2-SL1, loop deletion (19–21 loop deleted) and loop-proximal stem mutation (14–16GUA/19–22AUAC), as shown in Figure 3A. We observed that both SCOV1-SL1 and SCOV2-SL1 bind to both nsp1 proteins despite having different sequences in the loop-proximal stem sequence (Figure 3B and C). We noticed that SCOV1 nsp1 generates a larger slow-moving complex with both SCOV1-SL1 and SCOV2-SL1 RNA relative to the respective complexes formed with SCOV2 nsp1 (Figure 3C) even though individual proteins show the same gel mobility (Figure 3D). Since nsp1 of SCOV1 and SCOV2 form RNA complexes of different mobilities, we asked if SCOV1 nsp1 is present in an oligomeric form that is responsible for the slow-moving complex formed with SCOV1 nsp1 but not SCOV2 nsp1 (Figure 3B). To investigate whether SCOV1 nsp1 forms an oligomer, we purified GST-nsp1 (46 kDa) from bacterial lysate and incubated it with separately purified untagged nsp1 (19 kDa). Pull-down of 46 kDa GST-nsp1 on a glutathione column resulted in coprecipitation of 19 kDa nsp1, confirming oligomer formation (Figure 3D). However, SCOV2, which forms the faster-moving complex, also forms an oligomer. This result eliminates the possibility that the larger complex with SCOV1 nsp1 is due to dimerization of the protein. It is still possible that SCOV1 nsp1 binds multiple SL1 RNAs, resulting in a larger complex or binding to RNA changes the conformation of the complex resulting in a slower-moving complex. Indeed, after longer exposure, some of the nsp1-bound complexes are accompanied by additional bands that may arise due to conformation change of RNA upon nsp1 binding. Next, we seek to find if these RNA–nsp1 complexes will modify in the presence of cytoplasmic extract. Since viral RNA undergoes translation even in the presence of nsp1, we asked if these nsp1-bound viral RNAs can engage with ribosomes in the presence of nsp1 to participate in translation. We incubated RNA in the presence of nsp1 and added an increasing amount of cytoplasmic extract containing ribosomes.

**Figure 3:**
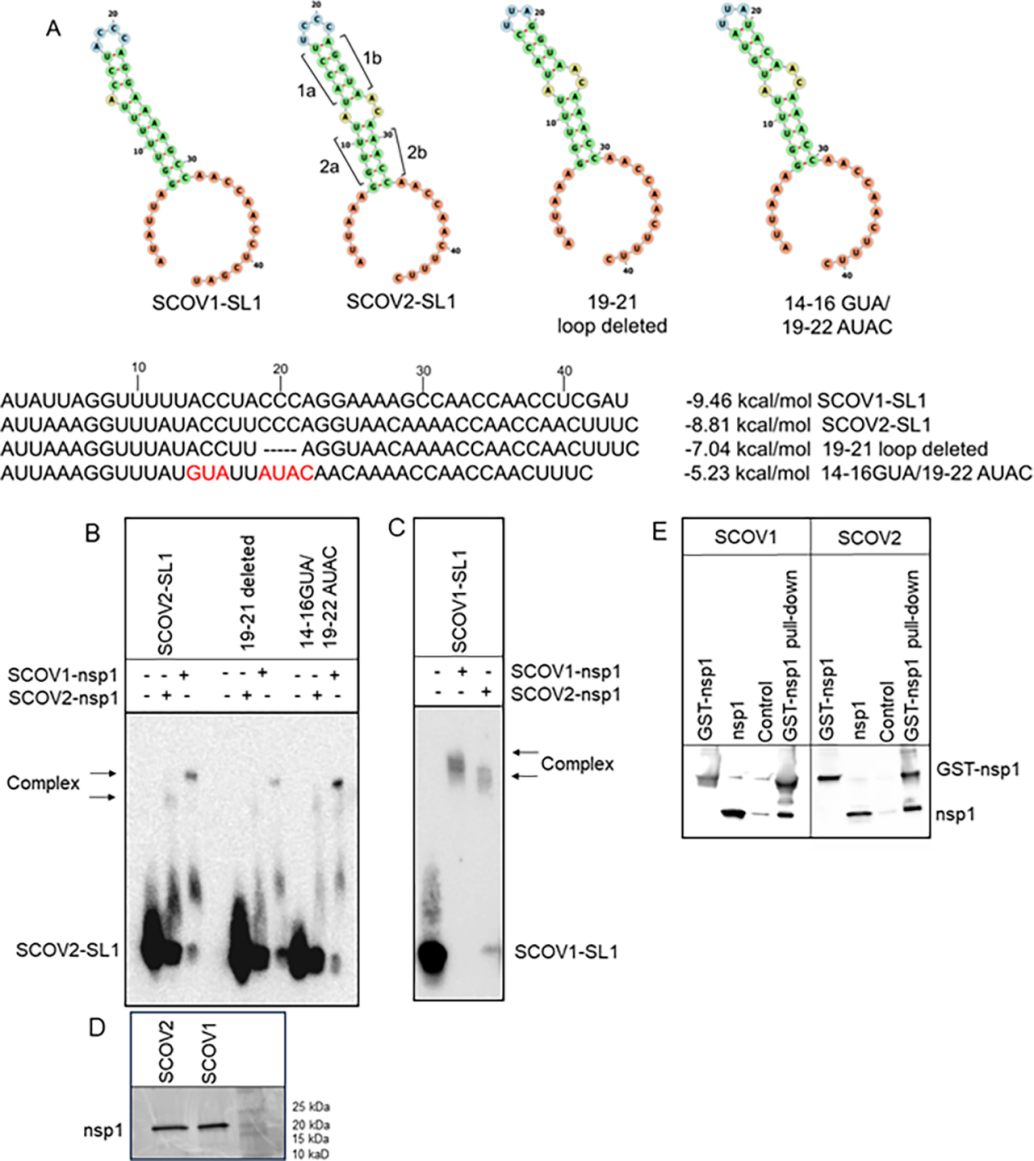
Both SCOV2 and SCOV1 nsp1 bind to SL1. (A) Sequence alignment and secondary structure analysis of SL1 of SCOV1 and SCOV2 along with SCOV2 stem-loop mutations in the RNA fold. (B) SCOV2 SL1 and stem-loop mutants bid to both SCOV2 and SCOV1 nsp1 proteins. RNA was incubated with 7 μM nsp1, followed by gel electrophoresis, transfer, and visualization of RNA complexes with chemiluminescence. (C) SCOV1 SL1 binds to both SCOV1 and SCOV2 nsp1 proteins (7 μM). (D) Coomassie staining of the denaturing gel with SCOV1 and SCOV2 nsp1. (E) Both SCOV1 and SCOV2 nsp1 proteins form oligomeric forms. The dimerization experiment is performed using separately purified GST-nsp1 (46 kDa) and untagged nsp1 (19 kDa). Glutathione bead-bound GST-nsp1 was copurified with nsp1, and complexes are eluted with glutathione. Complexes are analyzed by Western blot, and nsp1 is detected with the anti-nsp1 antibody.

Figure 4 shows that, in the presence of cytoplasmic extract, the RNA–nsp1 complex disappears, and two separate complexes form (one smaller and the other larger than the RNA–nsp1 complex), indicating components of cytoplasmic extract can replace nsp1 and form new complexes (Figure 4, lanes 3–5). Since cytoplasmic factors form a complex that is smaller than the RNA–nsp1 complex, we believe that this complex lacks nsp1. To understand if the larger shift in lanes 3–5 (Figure 4) is due to cytoplasmic factors alone, we also compared SL1’s ability to bind cytoplasmic factors in the presence and absence of nsp1 (Figure 4, lanes 6–9). Since the gel shift indicates multiple complexes with varied mobility, it is difficult to conclude if nsp1 caused additional gel shifts of these complexes. However, the presence of nsp1 enhances their formation, as shown by the higher intensity of these bands. These complexes may serve as precursors to successful viral RNA translation.

**Figure 4:**
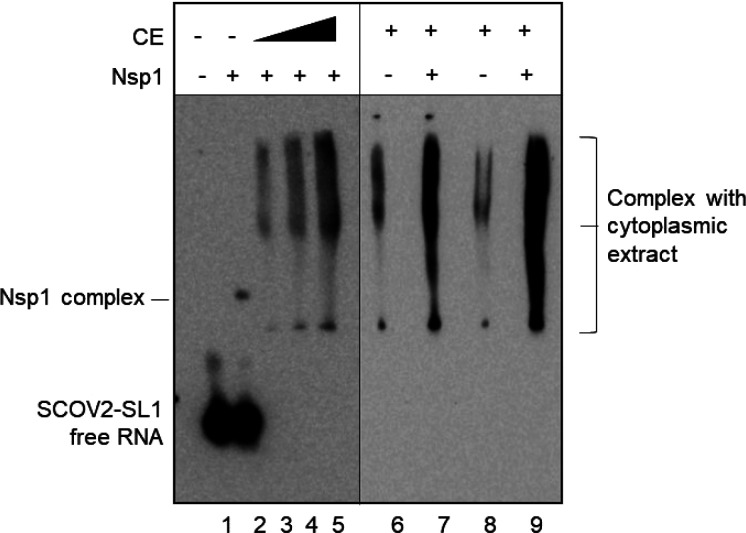
Viral leader RNA forms complexes with the cytoplasmic extract in the presence of nsp1. SL1 is incubated with nsp1 (6 μM). Lanes 1 and 2 show free RNA and RNA-bound nsp1 complex. Gel-shift assay shows the formation of a very small complex and several large complexes when the nsp1–RNA complex is incubated with the cytoplasmic extract (lanes 3–5). The RNA–nsp1 complex was incubated with 4 μg, 8 μg, and 16 μg of cytoplasmic extract (lanes 3–5). Lanes 6 and 8 are incubated with 8 μg and 16 μg of cytoplasmic extract only and lanes 7 and 9 have both nsp1 (6 μM) and cytoplasmic extract (8 and 16 μg, respectively).

Overall, our work demonstrates that SCOV-1 nsp1 binds directly to viral RNA in the absence of any bridging factor, but unlike SCOV-2 nsp1, SCOV1 nsp1 forms multiple complexes. The slow-moving complex forms in the presence of a high nsp1 concentration. In contrast to translation studies in SCOV2 with a mutated RNA leader sequence, mutation in the loop proximal sites of SL1 did not abolish binding by nsp1.^18^ These results indicate that either there is a separate requirement for nsp1’s binding to the RNA sequence and its ability to stall translation or sequence requirements for nsp1 binding in SCOV1 and SCOV2 are different. In accordance with studies done in cells expressing nsp1, these complexes are replaced by cytoplasmic components that may allow for viral RNA translation and virus propagation.

## Accession Codes

SARS coronavirus 1 nsp1 (1–180 amino acids) from Dr. Makino: AY274787.1. SARS coronavirus 2 nsp1 (1–180 amino acids) from Addgene: YP_009725297

## Abbreviations

SCOV1: severe acute respiratory syndrome coronavirus 1
SCOV2: severe acute respiratory syndrome coronavirus 1
nsp1: nonstructural protein 1
