# Supplementary material for: Characterization of nsp1 Binding to the Viral RNA Leader Sequence of Severe Acute Respiratory Syndrome Coronavirus
Source: Biochemistry. 2024 May 8;63(10):1235–40. doi: 10.1021/acs.biochem.4c00078 (PMC11112752; doi:10.1021/acs.biochem.4c00078)
Supplement: Supplementary file 1 — bi4c00078_si_001.pdf [file bi4c00078_si_001.pdf]

Supporting Information:

Characterization of nsp1 binding to the viral RNA leader sequence of severe acute respiratory syndrome coronavirus

Jonathan L. Cromer[‡], Laurie F. Melton[‡], Kaitlin M. Caughman[#], and Anita Nag*

Natural Sciences and Engineering, USC Upstate, Spartanburg, SC 29303, USA

*Department of Chemistry, Clemson University, Clemson, SC 29634, USA

[#]Harvard University, Cambridge, MA 02138, USA

KEYWORDS: SARS coronavirus, nsp1, RNA, viral leader sequence

Materials and Methods

Chemicals and Reagents

pGEX-nsp1 CoV1 plasmid (Urbani strain, accession no.: AY278741.1) and R124A/K125A and K164A/H165A mutants were kind gifts from Dr. Shinji Makino (University of Texas Medical Branch). pGEX nsp1 CoV2 was a gift from Dr. Britt Glaunsinger (Addgene plasmid # 175512). The anti-nsp1 antibody was purchased from ThermoFisher (Catalog no. PA116941). Biotinylated RNA was purchased from Eurofins and Integrated DNA Technologies (IDT). pGEX-nsp1 CoV1 was mutated to D33R, K58E, and R99E using QuickChange Lightning mutagenesis kit (Agilent) and oligos from Table 1.

Expression of GST-tagged protein

Wild-type or mutant GST-tagged nsp1 was expressed in BL21-Codon Plus (DE3) RIPL-cells (Agilent) in Luria Broth media. Cells were grown at 37°C to OD600 of 0.6 followed by IPTG induction (final concentration 1 mM). Expression was continued overnight at 28°C before spinning down the bacterial culture at 6,000 rpm. Cells were lysed using lysis buffer (5% NP-40, 0.3 mg/mL lysozyme) at room temperature for 30 minutes followed by a sonication (50% output, 30-second pulse 4 times with 30-second intervals). Cells were then centrifuged at 15,000x g for 30 minutes. The clear supernatant was incubated with glutathione beads (Genesee Scientific) containing 1 mM PMSF and protease inhibitor (Halt Protease Inhibitor, ThermoFisher) at 4°C overnight. Next, bead-bound complexes were washed with 20 bead volumes of GST binding and wash buffer (Genesee Scientific) six times. The GST tag was cleaved with PreScission protease (Cytiva) overnight at 4°C and nsp1 containing the supernatant was collected by passing through a polypropylene disposable column to remove glutathione beads. Protein was concentrated to a desired concentration using a 3K protein concentrator (Pierce). The purity of the protein was verified by SDS-PAGE gel electrophoresis.

Gel Shift Assay to detect RNA-nsp1 complex

Forty-four nucleotide long biotinylated RNA (6.75 nM) was first folded into the most stable conformation in a thermocycler at 53°C, 37°C, and 22°C for 5 minutes each followed by chilling down the folded RNA to 4°C. The binding reaction was performed by incubating biotinylated RNA with purified nsp1 (6-9 μM unless otherwise specified) for 30 minutes at 25°C following the manufacturer's protocol of Light Shift Chemiluminescent EMSA kit (ThermoFisher, Catalog no. 20158). The RNA complexes were separated in Novex 6% TBE gel (Invitrogen) and transferred to a positive-charge nylon membrane (Invitrogen) followed by incubation with HRP-tagged streptavidin probe and detection of chemiluminescence (ChemiDoc, BioRad) following the manufacturer's protocol. The cytoplasmic extract was obtained from the EMSA kit (2 mg/mL, ThermoFisher, Catalog no. 20158). The control biotinylated oligo was supplied with the EMSA optimization kit (5′-UCCUGCUUCAACAGUGCUUGGACGGAAC-3′, a sequence from *Pan troglodytes* ferritin heavy chain 1). The control RNA folds into a stem-loop structure (-6.83 kcal/mol) according to the RNAfold RNA structure prediction program.

Nsp1 oligomerization assay

GST-tagged nsp1 (46 kD) was isolated on glutathione bead and was incubated with separately purified nsp1 (19 kD) without the GST tag. Glutathione beads were washed six times with 20x bead volume of PBS buffer. The complexes were eluted with glutathione and proteins were run in a 4-20% pre-cast SDS polyacrylamide gel (BioRad). Proteins were transferred to a PVDF membrane followed by western blot analysis using an anti-nsp1 antibody (ThermoFisher, Catalog no. PA116941). The chemiluminescence image was captured using ChemiDoc Imager (BioRad).

Table 1: Mutagenesis DNA primers

Mutation	Primer sequence
D33R	tgcgtggcttcgggcgctctgtggaagagg
K58E	tgtggtctagtagagctggaagagggcgtactgcccc
R99E	agaaatggacggcattcagtacggtgagagcggtataacactgg
